# ‘Talking lines’: the stories of diagnosis and support as told by those with lived experience of rare forms of dementia

**DOI:** 10.1186/s12877-024-04988-1

**Published:** 2024-06-07

**Authors:** Samuel Rossi-Harries, Charles R. Harrison, Paul M. Camic, Mary Pat Sullivan, Adetola Grillo, Sebastian James Crutch, Emma Harding

**Affiliations:** 1 Dementia Research Centre, UCL Queen Square Institute of Neurology, box 16, 8-11 Queen Square, London, WC1N 3BG UK; 2 School of Social Work, Faculty of Education and Professional Studies, Nipissing University, Nipissing, ON Canada

**Keywords:** Young onset dementia, Non-memory led dementia, Family carers, Caregivers, Line drawing, Arts-based research

## Abstract

**Background:**

People living with, or caring for someone with, rare forms of dementia can encounter issues while obtaining a diagnosis and trying to access appropriate support. This can affect their wellbeing, quality of life, social relationships and employment status. This study makes use of an arts-based narrative approach to explore individual accounts of these experiences whilst also exploring how, in telling their stories, those affected by rare forms of dementia might invoke, and situate their stories in relation to, broader cultural narratives around dementia and illness.

**Methods:**

Semi-structured interviews were conducted via video-conferencing software with participants (*N* = 27), living with, or caring for someone with, a rare forms of dementia. Participants used line drawings to depict their journey from initial symptoms to the present day, followed by prompts to verbally narrate their experiences. All interview transcripts and line drawings were subjected to narrative analysis. Four sets of transcripts and drawings were then subjected to more in-depth analysis.

**Results:**

Analysis shed light on the struggles encountered by both care-partners and people with a diagnosis, while navigating a health and social care system that does not always understand their needs. This often led to individuals feeling isolated and unsupported. Accounts also depicted challenges to identity brought on by the process. The moment of diagnosis was also drawn in a complicated light. Individuals found comfort in gaining understanding, but felt fear at recognising upcoming challenges. Participants situated their own accounts against mainstream cultural narratives around what good support for cognitive impairment and dementia might look like, whilst also demonstrating the influential role they took on in pursuing the right care.

**Conclusions:**

The use of line drawing, alongside narrative interviews, allowed participants to tell complicated, sometimes anachronistic, stories about difficult experiences, whilst also reflecting on, and attaching meaning to, them. These stories highlighted pressing gaps in healthcare services and shone a light on the various pieces of collective action individuals were engaged in in order to improve them. Finally, in modelling some elements of the participants’ service provision which were working, the narratives pointed to future directions services might move in.

**Supplementary Information:**

The online version contains supplementary material available at 10.1186/s12877-024-04988-1.

## Background

### Rare forms of dementia

The term dementia describes a number of conditions with differing etiologies, all resulting in a progressive decline in cognitive function (Prince et al., 2015) [1]. Approximately 60–70% of dementia cases worldwide are associated with Alzheimer’s disease and, in most of these, the person affected is over the age of 65 (WHO, 2020) [2]. This common form of dementia tends, primarily, to initially affect an individual’s memory, thinking and the ability to engage in everyday activities (WHO, 2020) [2]. There are however numerous rare forms of dementia which are more likely to affect people before the age of 65 and which are associated with distinct and atypical symptom profiles, often being non-memory-led (Harvey et al., 1996) [3]. Some of these rare forms of dementia are underpinned by Alzheimer’s pathology - such as the visual syndrome posterior cortical atrophy (PCA) - whilst others are caused by non-Alzheimer’s pathologies. Behavioural variant frontotemporal dementia (bvFTD), is characterised by predominant changes in behaviour and personality including loss of empathy and socially inappropriate or impulsive behaviours (Piguet et al., 2011) [4]. Primary progressive aphasia (PPA) is a collection of rare forms of dementia in which impairments in speech and language communication are the leading symptom (Marshall et al., 2018) [5]. These rare forms of dementia also include those which are autosomal dominantly inherited, such as familial frontotemporal dementia (fFTD) and familial Alzheimer’s disease (FAD), meaning those with an affected parent have a 50% chance of developing the same condition (Greaves & Rohrer, 2019; Ryan et al., 2016) [6, 7]. These complex and atypical symptom profiles and features, alongside the younger age of onset and limited awareness amongst health professionals, can lead to a range of complex and unique challenges for the people affected, with often convoluted routes to diagnosis and significant challenges in finding support which is appropriately tailored to the symptoms, life stage and role changes they are negotiating (e.g. being of working age, existing family care commitments) (Millenaar et al., 2016; Harding et al., 2023; Roberts et al., 2023; Woolley et al., 2011) [8–11]. Further exploration and understanding of the ways both people with conditions, and informal care-partners experience these dual processes is therefore needed.

### Narrative approaches

Since the 1960s, the “narrative turn” within the field of the social sciences has led to the proliferation of research and clinical methods building on the principle that people organise their experiences and interpretations of reality in the form of narratives (Riessman, 2008; Murray, 2021) [12, 13]. Narrative research can incorporate postmodern, constructionist and realist epistemologies but all approaches are linked by a commitment to exploring not only the content of people’s stories but the narrative devices (e.g. plot, characterisation and genre) they deploy in telling them (Riessman, 2008) [13]. Murray (1997) [14] states that narrative approaches are particularly well-suited, within the field of health psychology, to exploring and producing accounts of illness which fall outside of the mainstream, and recent research supports this assertion. Werner, Isaksen & Malterud (2004) [15], for instance, used narrative analysis on qualitative interviews with women living with chronic pain, finding that the illness stories included detailed accounting of their suffering, whilst also incorporating the scepticism they experienced at the hands of both health professionals and members of the public. More recently, Rushforth et al. (2021) [16] used narrative interviews and analysis to examine the stories of people experiencing long Covid and found that they used narrative devices to produce persuasive accounts of their own poorly understood conditions, whilst also re-conceptualising their experiences and conditions as legitimate. Murray’s (2021) [13] assertion that storytellers use narrative devices to attach meaning to, and build a sense of identity around, certain experiences gives clues as to why narrative enquiry might be particularly adept at exploring atypical healthcare experiences. Rushforth’s et al. (2021) [16] study lends credence to this idea, in demonstrating how, through the act of sharing their stories, people experiencing long Covid were able to build a strong collective identity which in turn provided opportunities for collective action and advocacy. Another possibility as to why narrative approaches, particularly when applied to extreme or atypical cases, can help to expand theory around poorly understood illnesses, lies in Riessman’s (2008) [13] assertion that the act of storytelling allows individuals to draw upon, and situate their story in resistance to, broader cultural narratives around particular phenomena. Werner, Isaksen & Malterud (2008) [15], for instance, produced chronic pain accounts which incorporated, but also challenged, dominant narratives used by health professionals, about gender, pain and conceptualisations of what constitutes an illness.

Narrative methods used on their own, however, contain epistemological complications, such as: (1) the potential privileging of ‘good stories’ (e.g. those that are coherent, straightforward and have satisfying conclusions) over more disjointed or anachronistic ones, such as those often produced when individuals describe traumatic experiences, (2) the production of a story in a research interview with a narrative intended for an inherent researcher audience, and (3) individual narrators that are extracted from their lived social and political contexts (Bradbury, 2017) [17]. Building on this, interviews of any kind (narrative or otherwise) can pose barriers to participation for those with dementia with the demands they place on verbal expression and memory function, while innovative qualitative methods can offer unique opportunities for maximising participation and improving accessibility in research for people with dementia (Phillipson & Hammond, 2018) [18].

### Visual methods

Visual methods have been used for data collection across different methodologies for over a decade. These include, for example, photo voice (Bradbury & Kiguwa, 2012) [19], body mapping (Gubrium et al., 2016) [20], visual autobiography (Squire et al., 2013) [21], and spatial and identity mapping (Futch & Fine, 2014) [22]. One such methodology, line drawing, is particularly well suited to addressing the aforementioned limitations of a solely verbal narrative approach. Artists such as David Hockney have observed that compared to other visual methods, such as photography, “drawing takes time, a line has time in it” (Hockney, 2013) [23]. Berger (2005) [24] states that this slowness “questions an event’s appearance and in doing so it reminds us that appearances are always a construction with a history.” From a methodological standpoint, drawing also provides added space for self-reflection whereby both researcher and participant might receive, access and learn about “previously untouched experiences” (Brailas, 2020) [25]. Within an interview context, this dialogue might also help to build rapport between researcher and participant, whilst offering research participants a more interactive interview experience that better articulates their stories beyond words (Emerson & Frosh, 2004) [26]. Influenced by experimental artistic exercises, Harrison (2019) [27] endorses line creation, as opposed to representational drawing, as a means of increasing inclusion and accessibility, whilst also not excluding further self-expression or articulation if favoured by participants, while Crutch et al. (2018) [28] notes the potential of line-based art activities to be used in both research and public engagement.

### Study aims

The aim of this study was to produce and explore accounts of the poorly understood and minimally documented experience of diagnosis and support seeking amongst people living with rare forms of dementia, as well as informal caregivers. Two qualitative methodologies were employed in order to do so.

## Methods

This section presents information about participants, data collection, procedures and data analysis. The method, ‘Talking Lines’ (Camic et al. 2023) [29], was developed as a means of incorporating line drawings into a more traditional narrative interview format during data-collection. The second methodology, a form of narrative analysis informed by the theoretical work of Riessman (2008) [13], was chosen for its ability to explore two elements of the accounts produced, in particular:

1. The ways in which individuals invoked, and situated their stories in relation to, more mainstream cultural narratives around cognitive impairment, diagnosis and support.
2. The ways by which, in telling their stories, those affected by rare forms of dementia build a sense of identity around their experiences of getting a diagnosis and finding support.

Data was collected, using the aforementioned ‘Talking Lines’ protocol for line drawings, as well as narrative interviews. A full protocol paper outlining the steps and procedures of the Talking Lines data collection method has been previously published by Camic et al. (2023) [29]. The research was conducted in accordance with the COREQ 32-item checklist for interviews (Tong et al., 2007) [30] and a completed checklist can be found in the supplemental files.

### Participants

People living with a rare form of dementia (PLwRD), care-partners and bereaved care-partners from Canada (*n* = 15) and the United Kingdom (UK; *n* = 12) were invited to participate as part of a larger, international study investigating support for rare forms of dementia (Brotherhood et al., 2020) [31]. They were selected purposively on a basis of theoretical and maximum variation sampling (Ames et al., 2019) [32]. In practice this meant that researchers took into account representation of different types of rare forms of dementia, residence (United Kingdom or Canada) and lived experience (PLwRD, current family care-partners or bereaved family care-partners) to ensure a range of experiences and perspectives were represented. The inclusion criteria included: minimum age of 18 years old, can understand and speak English or Welsh, has the capacity to consent independently, and has access to the internet by computer, tablet or smartphone with a camera. Recruitment occurred via a larger multi-component study (Brotherhood et al., 2020) [31] where potential participants – members of an international support service for people affected by rare forms of dementia – were approached via an emailed newsletter. Those who expressed interest were sent additional information about the Talking Lines study. No participants dropped out.

Nine of the interviewees were PLwRD: primary progressive aphasia (PPA; 3), posterior cortical atrophy (PCA; 3), frontotemporal dementia (FTD; 1), behavioural variant FTD (bvFTD; 1) and a mixed diagnosis of younger onset Alzheimer’s disease and FTD (YOAD and FTD; 1). Eight participants identified as male, while 19 identified as female. Four participants were aged 25–44 years, 11 were aged 45–64 years, and 12 were aged 65–74 years. Eighteen participants were care-partners, 11 of whom had been bereaved when the interview took place. Eight care-partners had experience of FTD, five had experience of PCA, two had experience of YOAD, one had experience of PPA, one had experience of FAD and one had experience of bvFTD.

The project was approved by University College London Research Ethics Committee (reference 8545/004) in the UK and Nipissing research ethics committee (reference 10,233) in Canada. Consent was obtained through telephone or video interviews in accordance with the study’s ethical approval. For informed consent to be obtained, a form of clear affirmative action (positive opt-in) was required; this was not inferred from silence, pre-ticked boxes or inactivity. In addition, we adhered to Para. 26 (Declaration of Helsinki) which indicates that where obtaining written consent cannot be expressed in writing, non-written informed consent must be formally documented and witnessed. To achieve this, we recorded informed consent provided via telephone recordings and/or virtual face-to-face consent procedures via a secure and encrypted video platform (e.g. GoToMeeting) as outlined in Brotherhood et al. (2020) [31]. Ongoing determination relating to a participant’s capacity to consent in the UK was carried out in accordance with the Mental Capacity Act (MCA, 2005) [33] and MCA Code of Practice (Department of Constitutional Affairs, 2007) [34]. In Canada, the consent process is set out in the Tri-Council Policy Statement: Ethical Conduct for Research Involving Humans (TCPS, 2018) [35].

### Data collection and procedures

Twenty-seven participants were interviewed by researchers AG, SRH and EH during a 2-year period from June 2020 to March 2022. AG (PhD, female) was employed as a Research Assistant. SRH (MSc, male) was employed as a Research Assistant and EH (PhD) was employed as a Research Fellow. All interviewers had previous training and experience in qualitative, semi-structured interviewing. Participants had no previous knowledge of the interviewers except in EH’s case who, due to her role as a support group facilitator, had had previous relationships with a minority of the participants interviewed. EH recorded reflexive thoughts on her joint role as a “Researcher Clinician” in her diary and these were discussed regularly during the data collection and analysis procedure. In two cases, due to requests by participants, they were interviewed with a care-partner, yielding 25 interviews in total. All interviews were conducted remotely, and both video and audio were recorded, using the *GoToMeeting* (GtM) video-conferencing platform, with all participants taking part from their own homes. Prior to the interview, participants were sent drawing materials (pieces of firm paper card and blue pencils) in the post, along with a postage-paid return envelope. The interviews were semi-structured with a protocol stipulating prompts for the line drawings, but including opportunities for the interviewer to enquire about specific elements of the drawings or experiences described. Interviewers recorded field notes, in which they recorded technical issues, reflexive thoughts and any non-verbal gestures made by the participants, during and after each interview. A senior researcher and clinician (PMC, PhD) was present at all team discussions. He also listened to and commented on a sample of nine interviews to assess consistency and quality. Developing the protocol occurred over several years “with conceptual, material, and methodological aspects being field tested in different formats within support, research and public engagement environments” (Camic et al., 2023) [29].

The interviews included three practice prompts designed to ensure usability of the materials and technology: draw a straight line, draw any line that you would like, and draw a line from when you got out of bed today to show your journey around your home up until right now. These were followed by three main prompts: (1) Please try and think back to when you first noticed something was wrong; Draw a line that describes your journey from that point to when you were [your family member was] diagnosed, (2) From the time of diagnosis, please draw a line that describes your journey to that first time you accessed information and support, and (3) Since accessing support, please draw another line that describes your journey from that first time you accessed support to the present. These time periods were chosen in order to provide a focused chronological account of a part of the dementia journey not well documented nor understood by health and social care professionals (Millenaar et al., 2016; Roberts et al., 2023; Woolley et al., 2011) [9–11].

After drawing each line interviewees were asked to hold up their drawing to the camera in order for the interviewer to see what they had drawn and then ask questions about what it represented. During the joint interviews both the care-partner and the PLwRD were given the same interview prompts sequentially. Interview lengths ranged from 30 to 90 minutes. No repeat interviews were carried out. A decision to end data-collection after 25 interviews was made based on research team discussions about data adequacy and features that were intrinsic to narrative analysis research (e.g. Tanner et al., 2022) [36] and followed recent recommendations.

“that the consideration of the characteristics of the study at hand, such as the epistemological and theoretical approach, the nature of the phenomenon under investigation, the aims and scope of the study, the quality and richness of data, or the researcher’s experience and skills of conducting qualitative research, should be the primary guide in determining sample size and assessing its sufficiency” (Vasileiou et al., 2018) [37].

### Analysis

Narrative analysis, informed by the theoretical work of Riessman (2008) [13] and as applied by Tanner et al. (2022) [36], was used to analyse the interview transcripts. Riessman’s approach was chosen due to its focus on the interview context, the performance aspects and the narrative devices employed in the telling (Tanner et al., 2022) [36]. Riessman’s approach also foregrounds exploration of the relationship between the act of storytelling and the construction of an identity on the part of the teller (Tanner et al., 2022) [36]. Attention was paid to the ways PLwRD and care-partners, often with unique support journeys, incorporated broader social and cultural narratives around support, illness and independence into their own narratives.

All 25 interview transcripts, and sets of line drawings, were included in the first stage of the narrative analysis. The transcripts were analysed in the first instance by four researchers (SRH, CH, PC, EH) each working individually with a set of interviews, and narratives were discovered in a bottom-up process rather than being pre-identified before coding. No specialised coding software was used and all coding was undertaken using Microsoft Word and analogue methods (e.g. pen and paper). The unit of analysis comprised a combination of the interview transcripts and the lines drawn, in response to the three interview prompts outlined above. A ‘key observations’ template (adapted from Tanner et al., 2022) [36], including prompts and jumping off points under four headings: ‘Context (the site of telling/creating)’, ‘Thematic Content (What is this story about?)’, ‘Performance (How is this story told?)’ and ‘Development (How is this story structured?)’, was filled out for one shared transcript. This was followed by a team meeting to discuss each researcher’s analytic decisions and other relevant issues. Key observations were independently recorded for two additional interviews before another meeting to discuss preliminary ideas about recurring and divergent narratives. This process continued until the entire dataset had been analysed, with fortnightly meetings to discuss and review ongoing analysis. All meetings were audio-recorded and are available for review. Researchers also recorded any reflections or issues in a reflexivity journal and discussed them at team meetings. The line drawings were also included in this procedure. Researchers systematically recorded terms and vocabulary used in the interviews to describe different components, shapes or styles of the line drawings, alongside the experiences and feelings these represented for participants.

Once key observations were recorded for the entire dataset, all four researchers met in person to develop an initial analytic framework (See Supplementary Table 1, Additional File 1). The team also analysed the line drawings during this meeting. Common methods and forms were identified, such as flat or straight lines, lines going up or down, waves, spirals, symbolic, figurative, mapping or metaphorical approaches. These elements were further organised across participants, after the meeting, by one researcher (CH), who overlayed text samples on top of the drawings to compare how different shapes were used and how they related to the words spoken in the interviews. This process led finally to the creation of a common reference document, called the narrative visual glossary. An abridged version of this document can be found at the top of the results section (Table 1).

**Table 1:** Visual Narrative Glossary (abridged)

**FLAT / STRAIGHT / LEVEL** - Even keel (pre-diagnosis) - Stability / calm	**UP / RISING / SWOOPING** - Gaining understanding / learning - Increasing depression	**DOWN / FALLING / DIPPING** - Diagnosis / direction change - Incorrect diagnosis / hopelessness / no future
**SPIKES / ZIGZAGS / JAGGEDY** - Big emotional, psychological incidences / trauma / violence - Extremes / manifestations	**PEAKS / VALLEYS** - Narrative turn / improving after decline - Symptom uncertainty / not knowing	**WAVES / SPIRALS / LOOPS** - Rapid uncontrollable decline / hopelessness - Care getting harder to manage
**STEP DOWN / STEPS / BABY STEPS** - Negative realisation - Progression / increased support need	**FIGURATIVE / METAPHORICAL (Black boxes / Little pills)** - Diagnosis / Stuck without knowing what’s next - Medication	**BODILY (Hands / Hearts)** - Connection with others / wanting peaceful solution - Changing health / physique
**TEXT / PUNCTUATION (Question marks / Names)** - Uncertainty / what to do? - Unusual symptoms / conflicting information	**MAPPING / DIRECTIONAL / ARROWS** - Uncertainty about how best to represent experiences - Creating linear, temporal chronology	**ABSENCE / METHODOLOGICAL (Resistance / Dissatisfaction)** - Method interpreted as restrictive - Regretting placement

Bold text denotes forms used. Plain text denotes common representational uses, as described by participants. Note: For an unabridged version, see Supplementary Table 2, Additional File 2

Based on the process outlined by Tanner et al. (2022) [36], four of the original participants were then selected for a more in-depth analysis, guided by both the initial analytic framework and visual narrative glossary. The four interviews entered into this round of analysis (and reported on below) were chosen, through consensus, by the four researchers conducting the analysis. All names appearing in the document are pseudonyms. The decisions as to which transcripts to include were based, again drawing on Tanner et al. (2022) [36], on two major criteria: (1) that they represented an even split between people living with a rare form of dementia and care-partners, and (2) that their accounts, when combined, were broadly illustrative of the main narratives contained within the initial analytic framework.

## Results

This section will outline three sets of results, stemming from both the first and second rounds of analysis. The first of these is an abridged version of the narrative visual glossary (Table 1), created during the first round.

The second set of results is a table of the narrative themes and sub-themes (Table 2), followed by a breakdown of the major narratives themes therein. These were refined and finalised, based on the initial analytic framework (See Supplementary Table 1, Additional File 1), during the second round of analysis. This process largely involved researchers removing or combining certain sub-themes, in the interest of concision and clarity. The biggest change between the initial analytic framework and the final Table was the decision to remove one narrative, “Covid as Disruption (sub-narratives: Lockdown as an Interrupter/Derailment, Derailed Ability to Give back to the Community, Forced to Face up to/Manage Situation). This decision was made on the grounds that it represented a common plot point, present across many individual accounts, but that the narratives included within it were often extremely context specific, and therefore had limited transferability across other contexts or time points.

**Table 2:** Narrative Themes and Sub-Themes

**Keeper of the Torch** Alone and Unsupported Maintaining Identity Seen Past Diagnosis Relational Knowledge	**Taking Action** Going Against the Grain It’s Up to Me Speaking Out Peer Support	**Detective Stories** Symptoms Accumulating Dealing with Professionals Diagnosis Double-Edged Lost in the Desert
**Climbs and Falls** Emotional Rollercoaster Responses to New Information Was Support Appropriate?	**A New Reality** Our Life Interrupted Accepting and Confronting Changes Looking to the Future There is no Cure	

### Summary of narrative themes

#### Keeper of the torch

Expressing feelings of aloneness as well as responsibility for preservation of self, or the selfhood of another, in the face of challenges brought about by symptoms and diagnosis.

#### Taking action

In the absence of support or resources from health and social care, or the charity sector, participants address, or speak out about, those gaps.

#### Detective stories

Trying to find answers or guidance from healthcare professionals in a landscape of accumulating unexplained phenomena or changing symptoms.

#### Climbs and falls

Support journeys were often characterised by turbulence, unpredictability and a continual need to recalibrate and adapt.

#### A new reality

Coming to terms with the realisation that life has changed and that the future looks dramatically different or uncertain.

### Participant accounts

The final set of results are the in-depth narrative accounts, and accompanying line drawings, produced during the second round of analysis. These log the stories of four participants: Amanda, Helen, Julie and Diane.

### Amanda

Amanda (female) was in her early forties, and caring for her mother who was in her sixties and in the advanced stages of familial Alzheimer’s disease, at the time of interview. Amanda had also undergone genetic testing herself which revealed that she too carried the same mutation. This meant she, too, would go on to develop the same condition. Amanda cared for her mother at home after returning from living overseas where she had been working as a nurse.

**Fig. 1:**
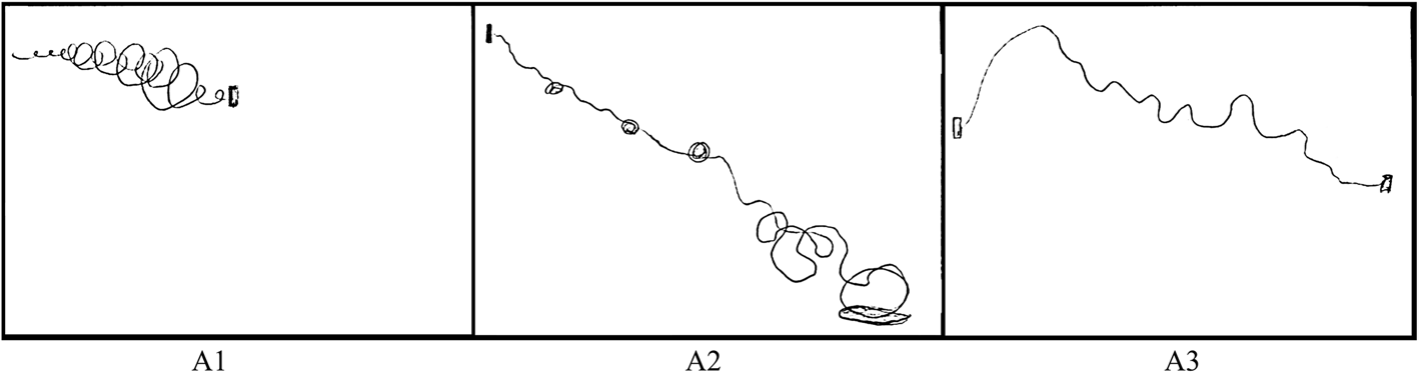
Amanda’s Line Drawings

Within her interview, Amanda described an initial period of increasing uncertainty as her mother’s symptoms developed and as she became aware of these changes from afar. This caused her to embark on a search for a diagnosis and her retelling of that period took the form of a *Detective Story,* characterised by many “*bumps in the road*” (Fig. 1, A1). The interview context offered a vantage point of hindsight, allowing Amanda to clarify the changes she experienced and what they went on to mean. This insight was not available to her at the time. Amanda’s experiences of increasing difficulties were captured, in her drawing, as circles of increasing size (Fig. 1, A1). During the interview itself, she chose to narrate the experience from her mother’s perspective.

*It just became harder and harder for her to live and getting, I guess, scarier and scarier for her when she didn’t really understand what was happening to her.*

This performative perspective shift contributed to the *Keeper of the Torch* narrative Amanda had been outlining throughout her account. In this narrative, Amanda was charged with *Maintaining Identity* on behalf of her mother and in doing so, enabling her to be *Seen Past Diagnosis*. Aside from the performative aspect, this tension was also evident in the contents of Amanda’s story. She made multiple references to mother’s characteristics before the diagnosis, and also often, in the following passage, contrasted her mother’s symptoms with more typical presentations of dementia in older people.

*Trying to take mum to a day care centre where there were people in their late 70s and 80s... She wanted to...touch everything and hold everything and... She wanted to go for a walk. She didn’t want to sit down with all the old people.*

The experience of witnessing her mother being *Seen Past Diagnosis* was also constructed, within her telling, as a marker of good examples of support.

*Mum could be whoever she was that day and you didn’t have to explain. Mum wanted to go round banging the chairs or smashing things or… If I’m feeding her tea and biscuits, that was okay. Nobody gave you a second look.*

Amanda described the diagnosis as a “*roadblock*”, and depicted it by drawing a solid black rectangle (Fig. 1, A1). This created a sense that their journey beyond that point was unknown and contingent on the support that would be available. Much of Amanda’s narrative beyond this point described *Climbs and Falls,* due to inconsistencies in support at multiple levels.

*People say, “Oh well, these people can come and do a sit-in or these people can… Phone this number and you’ll get this help or…” You don’t really get any help. There’s no one that’s, physically, helped us, it’s just been empty promises along the way really.*

The support inconsistencies described included discrepancies in the provision, availability, appropriateness and continuity. She also described inconsistencies in quality of care offered by broader systems — her experience with these was often poor — and contrasted this to the support provided by smaller organisations or individuals. These latter examples of support were often described as more helpful and supportive, with one depicted visually as a *“cushion”* which acted as a *“soft landing”* (Fig. 1, A2).

*When I first met the charity, yes… I guess it felt great to actually feel like there’s a place for us.*

However, even in describing more consistent support, Amanda evoked a sense of precarity, worrying that it would not be sustained. The general sense of inconsistency evoked helped contribute to an *Emotional Rollercoaster* narrative with many ups and downs reported throughout the interview.

*It’s just the ups and downs, really. Sometimes you feel like you’ve found somebody that’s having a positive influence but the consistency isn’t there so they disappear.*

It is also worth noting that Amanda’s line drawing depicted her support journey as having an overall downwards trajectory (Fig. 1, A1/A2/A3). While this account was often interrupted by loops, spirals and circular stopping points, symbolising various support groups and information received, the overall account seemed to capture the pervasive sense of *“exhaustion”*, with Amanda noting that her pursuing support led to *“a lot of dead ends”*.

The downwards trajectory (Fig. 1, A1/A2/A3) within Amanda’s account seemed also to represent other aspects of Amanda’s experience, including her mother’s symptom progression, her own energy levels decreasing, as she spent *“more time chasing up things that [weren’t] going to go anywhere”*. It also seemed to depict her mental health more broadly, as she experienced an *“ongoing spiral of emotions”*.

Much of Amanda’s account captured a sense of the responsibility (partly shaped by her nursing background) and aloneness she felt, whilst seeking support for her mother. While it featured many instances of her *Going Against the Grain* and *Taking Action,* suggesting a sense of agency and progress, it was clear, also, this experience was an *“ongoing struggle”* for her. This struggle was seemingly exacerbated by a feeling of being alone. We can see this in the way she positions herself and various health and social care professionals as working towards different outcomes, and coming from different perspectives:

*They’re [Professionals] asking me if I’ve tried to give mum a cup of tea – she didn’t need a cup of tea at 1 o’clock in the morning when she was screaming the place down, pulling at the curtains. She was scared, terrified, and needed more medication.*

*Nobody would look after mum because her behavioural issues were so bad...It’s better when it is just me and mum, to be honest.*

This sense of being *Alone and Unsupported* was also reflected existentially, in Amanda’s description of the uniqueness of her broader situation due to the genetic nature of the dementia within her family. The complexity of Amanda simultaneously being a care-partner, as well as a pre-symptomatic mutation carrier, for the same disease, creates a dual role for Amanda. This, in turn, complicated her experience of facing the future. For example, Amanda described the challenges, and sense of isolation she felt, when joining support groups as a care-partner, whilst knowing that she would go on to become a person living with dementia.

*It is really good having these groups but, in another way, it can feel more isolating because there isn’t actually anybody that I’ve found in the same position as I am.*

This was also captured in the development of Amanda’s story. As the interview progressed, she increasingly referenced a time beyond her mother’s support journey with projections into the future relating to her own imagined support experience.

*I know what I’m trying to give mum and I want somebody to try to do that for me too...seeing what’s out there just terrifies the life out of me getting it when it’s my turn.*

### Helen

Helen was in her mid 50’s and was interviewed at home where she lived with her husband, her pets. They had two children who had left home but both lived locally. She received a diagnosis of bvFTD 4 years prior to the interview, after a series of assessments with medical professionals. Before her diagnosis, both she and her husband had been working full-time, but Helen had since had to stop working and driving.

**Fig. 2:**
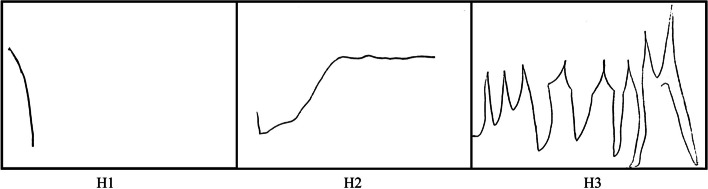
Helen’s Line Drawings

The interview showed an interaction between *Keeper of the Torch* and *A New Reality* narratives, although aspects of *Detective Stories, Climbs and Falls* and *Taking Action* were also present. Helen articulated attempts to preserve her changing identity in relation to her husband, medical professionals and others living with dementia. Before diagnosis, she explained how she had “*done my same job for years in a school office and I was just getting a bit muddled with some things. It was a bit odd, just something wasn’t right, feeling right.”* She described the steep drop from the left edge of her drawing before diagnosis (Fig. 2, H1) as *“just very low feeling”*, *“driving to work feeling like I wanted to end my life every day. It was ridiculous”.*

This despairing situation was expanded on, as Helen performatively recounted a story of the day she received a diagnosis. In the passage Helen presented herself as a character, using speech, asides, and the humour of a coincidental video recording to recollect that devastating moment in time. The story highlighted the confrontation between her seemingly illusionary hopes for *Maintaining Identity* and the interruption brought about by *A New Reality* of a diagnosis for her and her husband.

*“The day of my diagnosis is ever so funny because I don’t know why, at the time, I did this little video of myself. I’ve still got on my phone saying to my husband, ‘Hi M (name of husband), I’ve had a phone call from’ […]‘And everything’s fine. My brain is absolutely fine. Nothing to worry about, blah, blah, blah, love you,’ you know, that, sort of, thing.*

*And then within no time, that’s when I found out, over the phone as well; over the phone, mind, one to one, not even with my husband at my side, that there was a diagnosis”.*

A feeling of being let down when *Dealing with Professionals* around the time of diagnosis was something Helen came back to many times throughout the interview, with Helen and her husband having to do a lot of detective work. Helen recalled how she and her husband *“knew everything wasn’t fine”* even when professionals told them otherwise*.*

*I felt like it was almost as if I was making it up. That’s how I felt. I know that sounds silly. But, I thought, ‘Why don’t they believe what I’m saying?’*

The moment of diagnosis, after all of their joint detective work, was also recounted as *Double Edged*.

*I know it sounds ridiculous, but it was almost a relief when I got a diagnosis initially, to know what was going on, but then obviously afterwards, I thought, “Well, no, actually it’s not relief, is it?” It’s a bit stupid to think that.*

Helen’s recognition of the importance of *Peer Support* and the process of *Taking Action* to support others was evident in the way she described her post diagnostic journey, particularly in how she articulated the feeling of being overloaded with information and the comparative benefit of meeting with peers.

*Then I can remember getting loads of stuff in the post, loads and loads of leaflets, ridiculous amount of stuff.” […] “I honestly think the most helpful thing to people and their partners with it is to meet other people in similar situations because they’re the ones that can advise you on all these different things.*

The practical help Helen and her husband received from peers led to Helen *Taking Action* in her community and acknowledging the range of experiences of people in their situation.

*I liked doing that, being involved as much as I can to do things, to raise awareness, especially for the friends I’ve met with far worse situations than me.*

This consideration of, and connection with, others had a positive effect on Helen, evidenced in the stability and flatness of her drawn line (Fig. 2, H2) which Helen described as *“feeling better and feeling like me”*.

Sadly, the value of peer support for stability and *Maintaining Identity* was complicated by the COVID-19 pandemic, and the resulting drawing (Fig. 2, H3) showed steep, sharp *Climbs and Falls* and a rollercoaster of emotional ups and downs. Helen communicated that the ups in this drawing represented moments when she was active or enjoying time with other people. This, however, was contrasted with the difficult downs: “*But the low days, as well, as the line is down. They just happen all of a sudden, I don’t have any control over it”*. The despair of the situation was also rendered vividly at the beginning of the interview: “*you can’t get out of your head. And it’s just, like ‘Oh, I’d be better off if I wasn’t in this world.’”*

This challenging and tumultuous situation was expanded upon in a further story, which gave deeper insight into Helen’s difficulties with maintaining and understanding her changing self. This was possibly related to her specific diagnosis (bvFTD), leaving her feeling *Alone and Unsupported*. The story also highlighted the potential tension and strain this might cause to her relationship with her husband.

*“I get told off sometimes for being too out there saying everything I think, […] My husband says to me, “[Helen] you can’t do that all the time now.”*

Helen, at the time of the interview, seemed to have been finding things difficult, particularly in confronting her dementia diagnosis and *Facing an Uncertain Future*. Her friends, family, pets and peers contributed greatly to a positive and stable mood, and although Helen twice articulated deep discomfort with herself in the world, towards the end of the interview, she highlighted a key resolution and action towards her future and *A New Reality*, *“I just think, I’m not giving into it, I like to keep as active as I can and as busy as I can.”*

### Julie

Julie was in her early sixties and living in the north of England with her partner at the time of interview. She had received a PCA diagnosis 6 months previously and first noticed symptoms (a loss of ability in her left hand) 3 years prior to that.

**Fig. 3:**
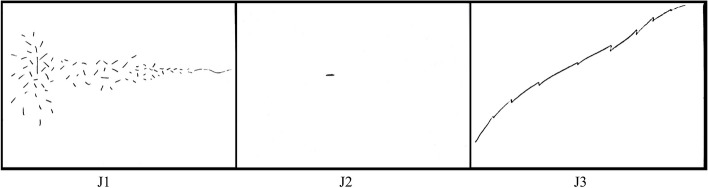
Julie’s Line Drawings

*Yes. It was like an explosion. It was not an immediate explosion, over a period of maybe a year or so, where all this was happening that I couldn’t understand what was happening. And then, as the journey progressed, with the assessments, and everything, I started to get a little bit of understanding, and then the huge relief […] when I got the diagnosis. It would have been better to have found there was nothing wrong with you […] And losing all my abilities for anything administrative, or numeracy, some literacy […] I’ve been self-employed for most of my life, all the things that I pretty much lost, was everything I needed. […] that’s what those little lines represent, with all these little things that came together, to make this big thing of the build-up. And then, when I got the diagnosis, to be relieved to understand. *(Fig. 3, J1)*.*

Here, Julie described an accumulation of unexplained symptoms, knowledge gained from professionals and finally an explanation, in the form of her diagnosis, typical of *Detective Stories*. Julie’s relief and disappointment at the diagnosis also exemplified the *Diagnosis Double-Edged* theme.

The passage, along with Julie’s assertion that she has been self-employed “*most of [her] life”,* also displayed elements of *Burden of Maintaining Identity*, particularly in how it dealt with Julie’s perceived loss of ability and the ways this impacted her work*.* Julie losing certain abilities didn’t just raise questions. It contributed to a shift in her self-concept.

Julie’s preoccupation with the loss of abilities pertinent to her job came up multiple times elsewhere. She took care to rephrase the prompt, “*think back to when you first noticed that something was wrong*” to “*when I first noticed things were going wrong with my abilities*” before drawing her line. The sense, within Julie’s account, that her lost abilities were connected to her fragmented sense of self was also rendered in the line drawing (Fig. 1, J1), in her own words “an explosion” of “little lines” which eventually came back together at the point of diagnosis. Julie even framed the interview context, the act of line drawing itself, as symbolic because she had to use her right hand.

Thinking in terms of how Julie performed her own story, it was evident that her concern with maintaining elements of her own identity affected the way she described her experience.

*Because I’m a very curious individual, and I ask a lot of questions, and that’s the way I’ve been all my life.*

Here, she explained how her own self-concept as a curious person gave her a sense of agency during the diagnosis seeking process. Julie then went on to narrate the *Climbs and Falls* of her post-diagnostic journey, whilst continuing to incorporate elements of *Keeper of the Torch*.

*The support has gone and grown from sitting with Doctor X and him actually saying, “I’m going to do this for you” […] But you’re so sceptical in life now. You think, “Oh, yes” […] this will happen in- Christmas is coming up, people are on holiday, but I thought, “If somebody rings in January, that will be fantastic” So, to find from talking to him, within several days, before Christmas, we’ve started that climb.*

This passage contained one of many instances wherein Julie portrayed her rare form of dementia journey as atypical. She was pleased to find her expectations around waiting for support subverted. The short timeframe was also rendered in her line (Fig. 3, J2), something she prefaced by saying “*you won’t believe this”* to the interviewer*.*

*…and the whole thing is just continuity all the way through. So, I feel, from what I’ve heard from others, I won’t say I’ve been exceptionally lucky, but it feels like I’ve been exceptionally lucky with that support that I’ve had, with information.*

Here, Julie drew again on her understanding of rare form of dementia support journeys, marked by a lack of continuity, before illustrating the atypical nature of her own experience. In this way her narrative both evoked a received narrative around inadequate support structures (that Julie was “*lucky*” to have fared better), whilst modelling a preferable alternative.

The narrative theme *Seen Past Diagnosis* was also prevalent in Julie’s account, and in a way which spoke directly to Julie’s *Burden of Maintaining Identity* during her pre-diagnosis journey. The pre-diagnosis journey represented 3 years, but was rendered without much detail. For example, Julie’s interactions were described as “*assessments and everything”*. Here, however, despite the timeframe being shorter, time seemed to dilate during Julie’s telling. She met with more professionals, doctors, an occupational therapist and staff members at two dementia charities, but these interactions were rendered as detailed scenes with distinct characters, lots of reported dialogue and inner monologue.

*So, she has helped with […] practical solutions, but also […] the mental side of things, but that side of things where she will say to me- she worked me out quite quickly.*

*Race along at 100 miles an hour, get jobs done, bit of a perfectionist […] she put it to me was, “It’s a journey. And we’re going on this journey together. And let’s say we’re at the railway station. Instead of hopping on the next train, why don’t we hang around for a bit, and have a cup of tea.”*

Julie ended her story on a hopeful note, bringing attention to her present situation. She used her line drawing (Fig. 3, J3) to indicate that she was still experiencing life as a series of *Climbs and Falls* but highlighted, also the important support she received, from various professionals, in making sure those challenges didn’t become overwhelming.

*Those little hooks are actually depicting me […] obviously, on this journey, on this mountain that I’m climbing where I’ve got the support, and we’re getting- I’m not saying we’re near the summit, but we’re starting to see the lovely bright sunshine, and the snow-capped tops. This is me having my days of, “Oh, goodness me” […] that’s not me wallowing, by the way, with anything, in any way.*

*But to show a truthful line, it’s not a straight line, but it’s a climbing line because all of you guys, every single one of you, excuse me, have actually kept me on the mountain path. So, I haven’t strayed too far off-piste.*

### Diane

At the time of the interview, Diane, in her mid-50s, lived at home with her husband. Married for nearly 30 years, they have no children from this relationship. He began experiencing symptoms about 3 years ago and was subsequently diagnosed with young onset Alzheimer’s disease after 2 years of multiple healthcare assessments. Both had been working full time prior to the diagnosis.

Shortly after taking early retirement, her husband became abruptly aware of symptoms when travelling alone on two flights. Calling Diane from the airport she reported, “*He was crying. He was upset. He couldn’t cope with doing that, even though I had set everything up for him. I had coached him along via text messages and calls*”. This travel day was noted as the beginning of the *“intense”* emotional *Climbs and Falls* that continued to occur for both of them. This sense was also reflected in her verbal and visual telling of her story.

**Fig. 4:**
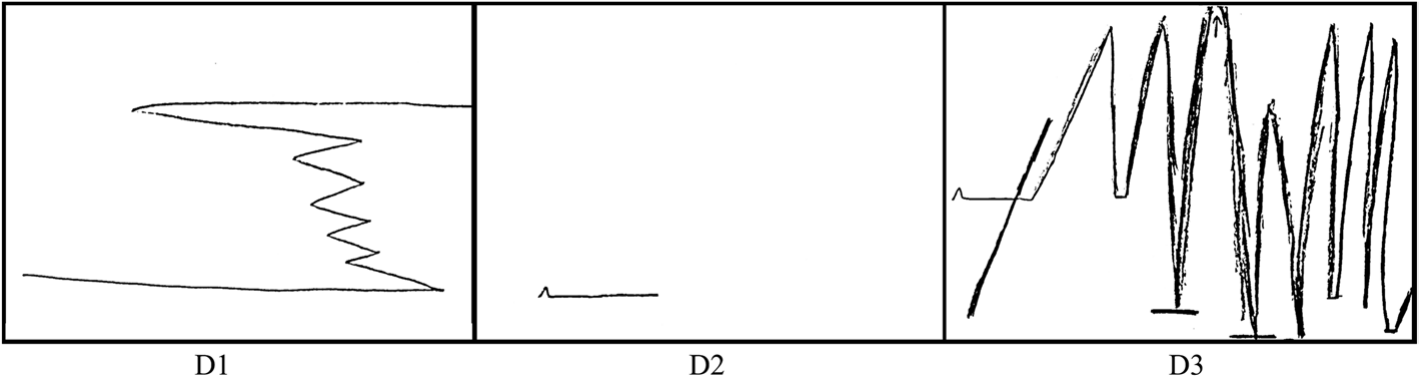
Diane’s Line Drawings

*“Now in retrospect I might have written a line straight across, sort of indicating even keel, you know, excited about the future. When I started noticing changes, there was one incident in particular that just boom, I just dropped right to the bottom, almost as far as I could go.”* (Fig. 4, D1).

When asked more about her line, Diane said “*The first indicator is the line up, it was more relief, him admitting and wanting to seek help*.” From childhood onwards Diane described herself as someone who “*tries to figure things out on my own*”, which has been a key component of *Maintaining Identity.* Asking for support, she suggested, had always been difficult. Throughout Diane’s life, she relied on herself to organise and find solutions to things that came her way. Her husband’s acknowledgement that there was a problem allowed her to continue in a role that was familiar, but also include him at an early point in the dementia journey. This sense of inclusion and joint distress is most exemplified in Diane’s telling by her use of the pronoun, *“we”*. Referring to her drawing, Diane state that *“the ups and downs in that period would be, ‘Who are we going to see? Who are you most comfortable with seeing first?”* (Fig. 4, D1). In a moment of evaluation, she reflected as a carer how her husband’s symptoms had affected her, *“This is an indicator…No, I wasn’t as up and down. This indicates a lot of his emotional up and down which affected me.”* This distinction is important but often difficult for care-partners to acknowledge, and evidenced her ability to understand how her husband’s emotional reactions affected her.

Having to get things right, *A Keeper of the Torch*, often feeling *Alone and Unsupported*, was how Diane positioned herself in the absence of family or professional support where she *“took action”* and searched for a diagnosis in a healthcare system that provided no care pathways for the symptoms her husband was experiencing. *“I can keep it together”*, a familiar self-telling, whilst reassuring, resulted in increasing stress after the diagnosis began to sink in and the *Climbs and Falls* re-emerged: “*Then the plunge down again with, oh my God, yes, this is true. This is what we are dealing with*.” The diagnosis became a transformational crisis point, challenging her long held beliefs around needing to figure things out by herself.

*The first blip would be a mix of emotions. Almost elation that we can now move forward and figure it out. Then I move into action mode. We are going to do this, this and this. I’ve got the support. We need to ask people questions. We need to, I need to access…That’s the straight line. I didn’t make it very long because it’s not continuous for me, in the sense of I can’t keep strong all the time* (Fig. 4, D2).

Going forward after the diagnosis, and initially in partnership with her husband (again as exemplified by use of the pronoun, “*we*”), Diane suddenly slowed her telling, hinting at her vulnerabilities and becoming aware of limits to her resilience but also of the need to seek support. Here, interestingly, she started using the “*I*” pronoun more frequently. The healthcare context was also very much part of her story and seemed to shape her empowered, self-positioning: “*I access my people, because there is no navigation line or navigation system for this kind of diagnosis in our healthcare system in Canada… Here you are totally on your own.”* She used her line to depict the importance of her own action, when it came to seeking support: “*I think the straight line indicates my steadiness at reaching out, getting that initial support, whoever that might have been. I could forget about the ups and downs and simply stay on course for that period of time”* (Fig. 4, D2).

Pointing toward the arrow in Fig. 4, D3, Diane described her journey seeking support for herself and her husband over “*this last year [as] particularly difficult when here, for example, the stress level for me was unending”*.

*These are showing ups, downs, like super stressed dropping down to bottom. These sorts of low ebbs are indicators of my mood. Feeling hopeless. Feeling helpless. The sense of loss…the selfishness, you know, you shouldn’t feel that way…As well as I think the heavier lines indicate that I’m kind of in this by myself because he doesn’t have the capacity to understand. Yes, so there was a lot of ups, and my ups are not good ups* (Fig. 4, D3).

*A New Reality* of her role as a permanent caregiver hit her extremely hard, and whilst there was no resolution, Diane recognised she no longer needed to do it alone.

*The transition between, ‘Okay. This is our life.’ My role now is a caretaker for the rest of it. The hopes and dreams that we had together are no longer…But as my end line, it does not mean it is complete and it is put away. So this indicates sort of the up and down of going out of my comfort zone asking for help as well as the emotional piece that goes along with that. Yes, the emotional pieces of loss, grief, having to do it all, or thinking I do. Having to let that go.* (Fig. 4, D3).

Diane’s story depicted a person encountering many external and internal challenges across this period of time. This included engaging a healthcare system with little or no understanding of this type of dementia. Within this context she positioned herself, not as a heroine, nor as a victim, but as someone who increasingly realised that her identity as a care-partner was becoming permanent. Also, importantly, reaching out to others was not depicted as a weakness. This insight was a form of resolution for her, even though her journey continued to be one of both *Climbs and Falls* (Fig. 4, D3).

## Discussion

This study set out to collect and explore accounts of experiences of diagnosis and support amongst those affected by rare forms of dementia, paying particular attention to the way they invoked, and provided an alternative to, mainstream narratives around cognitive impairment. The narratives produced were striking in their depiction of people affected by atypical, challenging conditions, and tasked with finding answers and solutions in a diagnostic and support landscape that is not sensitive or tailored to their needs. This is evident in the creation of the ‘Alone and Unsupported’ and ‘It’s Up to Me’ narrative subcategories, and more specifically in Amanda’s account, which demonstrates these problems in detail, outlining a support journey marked by inconsistency and a preponderance of “dead ends”. The picture of the support landscape, as depicted by the participants, was not an encouraging one and whilst this is not the first study to demonstrate some of these issues (Millenaar et al., 2016; Roberts et al., 2023) [9, 10], the narratives produced gave a vivid account of the sense of burden, isolation and overwhelm the participants experienced in response to this set of circumstances. The stories produced were also striking in their ability to give personally meaningful accounts of individuals responding to a lack of appropriate support pathways, whilst simultaneously acknowledging the ways in which the storyteller’s experiences ran counter to traditional narratives around support and illness. Many of the ‘Taking Action’ narratives demonstrated this, depicting individuals both developing resilience in response to strife whilst also operationalising their distress in order to support others and affect change. Gabriel’s (2004) [38] writing about these kinds of illness narratives, talks about the complex exchange of authority and power that can take place when narratives created by those with lived experience of illness contradict those held by professionals with more generalisable and impersonal claims to authority and expertise. It may be that research of this kind is a potent means of legitimising the former kind of expertise in the eyes of those who hold the latter kind. Many of the people interviewed were seemingly aware of the educational potential of their own stories. ‘Speaking Out’ narratives, in particular, often took the form of a performance in which individuals showed an awareness not only of their immediate audience (that of the researcher), but of another potential audience, made up of peers and varied health and social care professionals.

While the majority of rare form of dementia narratives conformed to the above pattern, in which rare form of dementia experiences of support and diagnosis were defined by a lack of clear diagnosis and support pathways and a need for individuals navigating those processes to take on an empowered role in response to these shortcomings, one account in particular was striking in the way it situated itself in opposition to that pattern. Julie depicts a diagnosis and support journey which is both appropriate and timely, often to the storyteller’s own surprise. In this we can see how Julie situates her story in resistance to another orthodoxy, that rare form of dementia support will be inappropriate and slow in coming. Because of this, it is worth particular attention. For Julie, good support is both timely and involves professionals taking time to sit with her and acknowledge elements of her identity which may be neglected when seen through the lens of her new diagnosis. Studies have also identified timeliness as an important factor when it comes to support for care-partners of those living with both behavioural variant frontotemporal dementia and familial frontotemporal dementia (Tookey et al., 2022) [39], while the importance of individualised, tailored support has also been evidenced as important for both people living with, and the families of people living with, Primary Progressive Aphasia (Ho et al., 2023) [40].

The study also sought to explore Riessman’s (2008) [13] assertion that identities are assembled, dissembled and performed through storytelling, and particularly in the context of people affected by rare forms of dementia navigating the process of diagnosis and support. The narratives produced gave a complex and enlightening account of the challenges experienced by both care-partners and PLwRD in maintaining a sense of selfhood and identity. For instance, Helen described this challenge from the position of someone living with a rare form of dementia diagnosis, and Diane from the position of a care-partner. One striking aspect of both these accounts was the deeply relational component of maintaining identity and a sense of selfhood in this context, as each described confronting feelings of disconnection with those closest to them, and the ‘interruption’ to their lives with their partner. A study by Pozzebon, Douglas & Ames (2016) [41] has also identified the importance of support that recognises the individual and the need for relational identity. Furthermore, this struggle around identity and selfhood was evident both in the content of the narratives, but also in the way in which they were told. Amanda’s narrative, in which she embodies her own mother’s perspective whilst also giving an account of her mother trying to maintain a sense of identity despite a condition which threatens it, is a good example of this. We can also see in Julie’s line drawings an account of the threats towards her own identity through the appearance of certain symptoms, whilst also giving an account of the ways in which her diagnosis countered some of those threats. The importance of finding identity in diagnosis, despite a disease which challenges it has also been demonstrated, within the context of chronic pain, by Werner, Isaksen & Malterud (2004) [15].

### Considerations for research and practice

Participants willingly partook in drawing lines and responding to interview questions. This dual approach of line drawing during an interview encouraged an interactive process to occur between participants and interviewers that included clarification, reflection, exploration and at times, humour. The drawings also provided a visual representation of a temporal process across a mostly unknown and under-researched time period for people with different rare forms of dementia. There are multiple opportunities for further research within dementia care and support research as well as with other less well-known healthcare problems. Key stages across different, lesser known dementias are just beginning to be understood and documented (e.g. Crutch et al., 2017; Hardy et al., 2023) [28, 42]. Incorporating a line drawing component into future staging research would provide a visual representation of different stages to enrich understandings, facilitate meaning-making and also allow greater interactive participation by PLwRD (especially those with language-led dementias, such as PPA) and care-partners. The observation that participants were able to reflect on, and construct narratives about, illness experiences, both while still experiencing that illness and, in the case of bereaved care-partners, afterwards, also points towards future directions for research. Further work to explore, in more depth, the impact of these different timelines on participants’ stories - particularly on narrative elements such as finding resolution and the arc of the story – would be worthwhile and could provide valuable insights for understanding time-sensitive support needs. A separate study could, for instance, specifically explore experiences of support around bereavement within this population.

One unexpected output of the analytic procedure of the narrative visual glossary. Originally created as an ad hoc answer to two questions raised during analysis: (1) how to ensure researchers have a shared language for talking about what they were seeing in the lines and (2) how to include visual data within the study’s narrative analytic frame, the result ended up being a noteworthy result in itself, demonstrating the many ways in which participants used their lines. Some lines were more figurative, making use of symbols such as light bulbs, books and human figures, whilst others interpreted the task by creating lines depicting chronological events. There was also variation in terms of how people used and produced lines during the interview. Most tended to draw lines and then reflect on them, based on interviewer prompts, while some narrated their experiences as they drew or even went back to edit their lines after narration. This all speaks to the fact that the use of lines and focused storytelling to gather information about lived experiences places the narrator in an empowered position to tell and shape their stories in a very different way than questionnaires or interviews focused on ‘facts’, symptoms and treatment. Whilst it does not put the interviewee and interviewer on equal footing, it does allow a different tale to be told. At points during the interview both researcher and interviewee would engage in an exchange in which the interviewee would check whether they were allowed to use the line in a certain way. This collaboration, it could be argued, led to a more co-produced form of storytelling than had a solely verbal semi-structured interview had been taking place. Adding a storytelling component to quantitative research, including clinical trials, would provide more nuanced information and allow research participants to have more of a voice about their lives.

Methodologically speaking, the creation of documents, such as the visual narrative glossary, may be worth further exploration in research context. Whilst there was much crossover in the meaning encompassed by certain shapes or forms, there were also significant contradictions or opposing interpretations as to what one individual’s line might represent in comparison to another who used a similar form or term, perhaps highlighting the idiosyncrasy of individuals expression and openness of the method. The need for the deployment of interpretation on the part of the researchers, is at once a strength of the study and also an area that could benefit from further exploration. Further exploration of lines captured during data-collection wouldn’t necessarily have to be narrative or purely qualitative in nature. Hurtut et al. (2008) [43], for instance, has demonstrated the potential for using pictorial analysis to compare line drawings across a population. Further analysis of the lines themselves might make use of image analysis of this kind, alongside linguistic analysis of spoken data to compare, contrast, or to explore relationships between other aspects of verbal and visual storytelling.

It would be relatively straightforward to incorporate line drawings into initial interviews in clinical and dementia charity settings and residential care. Our experience has been that it takes very little additional time and economically provides a good amount of detailed information. Outside of care environments, exhibitions of line drawings (with proper consent) along with segments of verbal narrative, can be used to engage and inform the general public in discussions about rare forms of dementia.

Methodologically speaking, whilst the findings around shortcomings of the rare form of dementia diagnosis and support landscape are not novel, the narrative line drawing methodology gave individuals a unique set of tools to create a persuasive, chronological account of the ways in which this set of circumstances impacted them. The narrative method also allowed interviewees to construct themselves within their accounts, not as victims, but as the empowered protagonists of their own stories. However, whilst these kinds of “recovery narratives” can affect change outside of the research context and also be empowering for the participant within it, they should also, Rushforth et al. (2021) [16] asserts, be handled with care. Fetishisation of these kinds of narratives, in which heroic individuals find resilience and community in the face of a lack of diagnosis and support pathways, runs the risk of generating complacency on the part of clinicians and policymakers (Rushforth et al., 2021) [16]. One way to temper this potential to over emphasise the value in taking a stand against services which don’t meet one’s needs, is to pay particular attention to accounts, such as Julie’s, which model good support which doesn’t have to be fought for.

It is also evident that the narrative and line drawing tools gave interviewees a way to both construct and locate their own identity in relation to their diagnosis and experiences of support. It may be that the narrative approach is particularly well suited to carers and those impacted by a cognitive impairment which threatens identity, as it provides a set of tools through which to play out, and take control of, this particular rupture. It is also of note that a study by Harding et al. (2023) [8] demonstrates the ways in which the production, through shared experience, of a collective identity amongst those affected by rare forms of dementia has both therapeutic benefits and provides a platform for collective action.

One unexpected methodological finding was in the way in which a narrative approach based on the work of Riessman (2008) [13], particularly through its focus on exploring elements of people’s narratives such as plot and complicating action, proved adept at providing accounts of the aftermath of difficult life events such as a rare form of dementia diagnosis. The narratives comprising ‘A New Reality’, and Diane’s account in particular, demonstrated this, showing the ways in which distress is distributed, in the wake of a rare form of dementia diagnosis, amongst multiple people. It may be that marrying a systemic approach, which focuses on the ways distress is distributed relationally across a system of people “at points where significant and fundamental changes need to be made” (Dallos & Stedmon, 2013) [44], with a narrative approach bears fruit when it comes to thinking about research, intervention and psychological formulation for those affected by rare forms of dementia.

### Limitations

There are several limitations to this study that need to be highlighted. Narrative analysis, like most qualitative methodologies, does not claim to present generalisable findings for a specific population. In relation to the present study, our findings likely do not represent the experiences of all people who are affected by a rare form of dementia. The findings do, however, offer detailed temporally-situated information about “the circumstances in which narratives are put to use and attempt(s) to analyse the functions they serve” (Bamburg, 2021) [45] for a group of people who are often invisible to health and social care practitioners and policy makers. A second limitation relates to how data was collected. “Telling stories is a messy business” but research interviews tend to structure the context of storytelling to reduce this messiness in order to “shape the talk” and meet research objectives (Wiles et al., 2005) [46]. The decision to structure the interview based on three prompting questions may have altered or changed participants’ stories in ways we are not aware of. A third limitation is the retrospective nature of the study. Storytelling, which relies on memories, emotions and images from times past, may not always be able to be recalled. Yet recent research has found memories of past events, whilst malleable, are essentially reliable (Brewin et al., 2020) [47]. In addition, although care-partners of people living with dementia have found line drawing a helpful tool “to access unspoken thoughts and emotions and improve their understanding of non-verbal interaction” (McEvoy & Bellass, 2007) [48], it is possible that the addition of line drawing may have had an unintended inhibiting influence on some participants, or offered an inadequate format to communicate particularly complicated experiences or emotions.

The virtual nature of the interviews might also register as another limitation. It may be that further exploration of ways of applying this methodology in a non-digital setting, or even as a group activity, might provide new findings and opportunities for new ways of engaging with the task itself. People with significant visual impairment may not be able to, nor want to, participate in a drawing component as part of a research (or clinical) interview. One final limitation, with regard to the individual interviews specifically, was in the methodology’s reliance on asking for lines and stories from a single perspective. Dallos (2018) [49] has noted the potential for therapy which makes use of group narratives, and explores the ways a number of people tell a story, and from several different perspectives, as a means of enhancing systemic practice. It may be that further work using line drawing, in narrative interviews, could benefit from incorporating multiple perspectives, and even multiple lines, in their data-collection.

## Conclusions

This is the first study we are aware of to explore narrative accounts of both the diagnosis and support seeking amongst those affected by rare forms of dementia using visual line drawings. It offered insights into the challenges faced by these individuals, whilst also allowing opportunities for them to tell their own stories and, in doing so, construct a sense of agency and collective identity. It has also illuminated the ways in which arts-based, visual representations can aid those affected by rare forms of dementia in giving accounts of, and attaching meaning to, their experiences. Whilst the accounts highlighted many aspects of services in need of development during diagnosis and support seeking, they also detailed vital examples of ways in which services are excelling. Highlighting key principles of effective tailored support (e.g. that it is both timely & tailored to the individual) for these underserved populations can inform ways in which broader service provision might be improved.

### Supplementary Information

**Additional file 1.**

**Additional file 2.**

**Additional file 3.**

## Data Availability

The data that support the findings of this study are available on reasonable request from the corresponding author, [EH]. The data are not publicly available in compliance with ethical and funding requirements but will be uploaded to a data repository for researchers from different institutions to access after the study ends in 2024.

## Abbreviations

bvFTD: Behavioural variant frontotemporal dementia
FAD: Familial alzheimer’s disease
fFTD: Familial frontotemporal dementia
FTD: Frontotemporal dementia
PCA: Posterior cortical atrophy
PPA: Primary progressive aphasia
YOAD: Young onset alzheimer’s disease
