# Supplementary material for: ‘Talking lines’: the stories of diagnosis and support as told by those with lived experience of rare forms of dementia
Source: BMC Geriatr. 2024 Jun 7;24:504. doi: 10.1186/s12877-024-04988-1 (PMC11157747; doi:10.1186/s12877-024-04988-1)
Supplement: Supplementary file 1 — Additional file 1. [file 12877_2024_4988_MOESM1_ESM.pdf]

Supplemental Table 1. Initial Analytic Framework

NARRATIVE THEMES	NARRATIVE SUB-THEMES
**Keeper of the Torch**	Lost in the desert<br>We got inappropriate support<br>Not being defined by label (seen past diagnosis)<br>Alone and carrying weight
**Detective stories**	Diagnosis as a double edged sword<br>My/our experience is unique<br>Lost in the desert<br>I have to get this right
**The climbs and falls**	The push and pull of gaining knowledge & insight<br>We got inappropriate support<br>Care workers - costs vs benefits<br>Pathway to support (information overwhelm/information underwhelm)<br>We got good support<br>Seen past diagnosis<br>Plunge/dip/hole
**Taking Action**	I have to get this right<br>Performance/context in interview<br>Going against the grain<br>The encore (audiencing, speaking out, legacy),<br>I made things happen (agentic self positioning),<br>In the absence of support<br>Plunge/dip/hole<br>Community action/peer support/lifting others up
**Covid as disruption**[1]	Lockdown as an interrupter/derailment<br>Derailed ability to give back to the community<br>Forced to face up to/manage situation
**A New Reality**	No cure but what can you do?<br>Facing the future at the point of support<br>At what point do we accept things have changed<br>Grateful for the good things<br>Our life interrupted<br>It's made me who I am<br>Elliptical present - looking to the future<br>Elliptical present - taking every day as it comes<br>Not being defined by label (seen past diagnosis)

1. Different framing (Conflicting Action: often about a single event and its narrative repercussions, rather than an entire narrative in itself.)
