# Supplementary material for: ‘Talking lines’: the stories of diagnosis and support as told by those with lived experience of rare forms of dementia
Source: BMC Geriatr. 2024 Jun 7;24:504. doi: 10.1186/s12877-024-04988-1 (PMC11157747; doi:10.1186/s12877-024-04988-1)
Supplement: Supplementary file 2 — Additional file 2. [file 12877_2024_4988_MOESM2_ESM.pdf]

Supplemental Table 2. Visual Narrative Glossary (unabridged)

**FLAT / STRAIGHT / LEVEL**<br>- Even keel (pre-diagnosis)<br>- Stability / calm<br>- Acceptance<br>- Pause / stasis<br>- Feeling better / settling<br>- Access to info / relief<br>- Continuation of situation<br>- Stability / planning<br>- Calm (after difficulty)<br>- Getting support / less worry<br>- Support / safety net<br>- Lack of change / consistency	**UP / RISING / SWOOPING / CLIMBING**<br>- Gaining understanding / learning<br>- Increasing depression<br>- Things going well / managing outlook / momentum / support from others<br>- Harder challenges / Escalating symptoms / self-awareness / calmness<br>- Getting help / support<br>- Peer support<br>- Hope / direction / a way forward / information / knowledge<br>- Feeling understood and supported for first time<br>- Getting significant support (professional care)<br>- Support / coping<br>- Access to resources<br>- Safety / support / guiding<br>- Overcoming obstacles<br>- Gentle progress / getting better<br>- Resisting situation / gaining resilience	**DOWN / FALLING / DIPPING / CRASHING**<br>- Diagnosis / direction change<br>- Incorrect diagnosis / hopelessness / no future<br>- Emotional drop / realisation / embodied sensation<br>- Things not going well / relational decline<br>- Lowness / confusion / despair<br>- Degeneration<br>- Not knowing / knowing what was wrong<br>- (Negative) learning / anticipating future / Symptom progression<br>- Absence of support<br>- Uncertainty<br>- Immediate impact & reality of diagnosis<br>- Less upset / decrease in depression<br>- Challenge of finding information / seeking and accessing support<br>- Losing abilities<br>- Mourning
**SPIKES / ZIGZAGS / JAGGEDY / JERKY**<br>- Big emotional, psychological incidences / trauma / violence<br>- Extremes / manifestations<br>- Questioning / searching<br>- Figuring it out<br>- Key negative emotions / expression of concern<br>- Diagnosis impact<br>- Anxiety / stress / fear (relational)<br>- Practical response to Dx<br>- Super stressed / hopeless / helpless	**PEAKS / VALLEYS**<br>- Narrative turn / improving after decline<br>- Symptom uncertainty / not knowing<br>- Chaos & confusion pre Dx<br>- Constant changing situation<br>- Switching on and off to situation<br>- Mood fluctuations<br>- Having company & plans / sudden drops in mood<br>- Each stage / intensity of situations<br>- Lack of consistency in care & support<br>- Making progress / facing setbacks<br>- Progress / moving forwards<br>- Shared journey / maintaining contact<br>- Emergencies & crises / anger & exasperation	**WAVES / SPIRALS / LOOPS / BUMPS**<br>- Rapid uncontrollable decline / hopelessness<br>- Care getting harder to manage<br>- Confusion / uncertainty / worry / fear / sinking<br>- Long period of uncertainty<br>- Changing conditions<br>- Amount of supervision / help<br>- Gaining confidence / less agitation<br>- Overwhelmed / Internal turmoil / - - Starting to make sense of things<br>- Tangled emotions / muddled / day-to-day / no support<br>- Chasing / limited support / expending effort<br>- Managing despair<br>- Ground yourself in uncertain, chaotic situation<br>- Gentle acceptance<br>- Managing multiple setbacks<br>- Feeling deeply muddled

**STEP DOWN / STEPS / BABY STEPS**<br>- Negative realisation<br>- Progression / increased support need<br>- Moving towards getting help<br>- Managing each stage as it comes	**FIGURATIVE / METAPHORICAL (Black boxes / Little pills / First aid / Sun and clouds / Spectacles / Volcano / Explosion / Books / Lightbulb / Cushion / Hooks / Rocket)**<br>- Diagnosis / Stuck without knowing what's next<br>- Medication<br>- Anger / frustration<br>- Getting support<br>- Unusual symptoms / visual problems<br>- Eruption of emotion / reality<br>- Chaos & confusion / symptom impact<br>- Overwhelmed with information<br>- Comprehending diagnosis<br>- Supported but worried about future<br>- Lost / without direction<br>- Finding substantial support<br>- Recalibrating / readjusting<br>- Speed of medical input	**BODILY (Hands / Hearts / Faces / Brains / Tummy)**<br>- Connection with others / wanting peaceful solution<br>- Changing health / physique<br>- Fluctuating emotions and mood<br>- Hoping nothing changes / continuity of mind and self<br>- Holding each other in a relationship / sharing journey<br>- Family support
**TEXT / PUNCTUATION (Question marks / Names / Dates / Zero)**<br>- Uncertainty / what to do?<br>- Unusual symptoms / conflicting information<br>- Diagnosis answers questions<br>- Absence of information<br>- Shared journey together / highlighting key characters<br>- Partitioning different phases / highlighting key events	**MAPPING / DIRECTIONAL / ARROWS**<br>- Uncertainty about how best to represent experiences<br>- Creating linear, temporal chronology<br>- Structuring to represent multiple problems<br>- Unconventional choice of direction<br>- Different pathways of inquiry<br>- Highlighting intensity of specific moment	**ABSENCE / METHODOLOGICAL (Resistance / Dissatisfaction / Placement / Faintness / Thickness)**<br>- Method interpreted as restrictive<br>- Regretting placement<br>- Line not capturing experience accurately<br>- Inability to accurately depict intensity of feeling (fear & terror of future)<br>- Depicting emotion with pressure of mark-making (overwhelmed to easiness)

Bold text denotes forms used. Plain text denotes common representational uses, as described by participants.
